# Trend Shifts in Age-Specific Incidence for In Situ and Invasive Cutaneous Melanoma in Sweden

**DOI:** 10.3390/cancers13112838

**Published:** 2021-06-07

**Authors:** Hanna Eriksson, Kari Nielsen, Ismini Vassilaki, Jan Lapins, Rasmus Mikiver, Johan Lyth, Karolin Isaksson

**Affiliations:** 1 Department of Oncology and Pathology, Karolinska Institutet, 171 76 Stockholm, Sweden; 2 Cancer Theme, Department of Oncology, Skin Cancer Center, Karolinska University Hospital, 171 76 Stockholm, Sweden; 3 Department of Clinical Sciences, Dermatology, Lund University, 221 84 Lund, Sweden; kari.nielsen@med.lu.se; 4 Department of Dermatology, Skane University Hospital, 221 85 Lund, Sweden; 5 Department of Dermatology, Helsingborg Hospital, 251 87 Helsingborg, Sweden; 6 Department of Pathology and Cytology, Karolinska University Laboratories, 171 76 Stockholm, Sweden; ismini.vassilaki@gmail.com; 7 Department of Medicine, Unit of Dermatology, Karolinska Institutet, 171 76 Stockholm, Sweden; jan.lapins@sll.se; 8 Department of Dermatology, Skin Cancer Center, Karolinska University Hospital, 171 76 Stockholm, Sweden; 9 Regional Cancer Center South East Sweden, 581 85 Linköping, Sweden; Rasmus.Mikiver@regionostergotland.se; 10 Department of Clinical and Experimental Medicine, Linköping University, 581 83 Linköping, Sweden; 11 Department of Health, Medicine and Caring Sciences, Linköping University, 581 83 Linköping, Sweden; johan.lyth@liu.se; 12 Department of Clinical Sciences, Surgery, Lund University, 221 84 Lund, Sweden; 13 Department of Surgery, Kristianstad Hospital, 291 33 Kristianstad, Sweden

**Keywords:** cutaneous melanoma, age-specific incidence, trend shifts, joinpoint, average annual percentage change

## Abstract

**Simple Summary:**

The incidence of invasive cutaneous melanoma (CM) is increasing in Sweden. The aim was to present age- and sex-specific trends of the age-standardised incidence and the average annual percentage change (AAPC) for in situ and invasive CM by analysing data obtained from the Swedish Melanoma Register and the Swedish Cancer registry for 35,350 in situ tumours and 59,932 CM. Trend shifts in age-specific incidence for in situ and invasive CM reflect a rise among both sexes since the 2000s and could be a result of more effective secondary prevention efforts and a higher awareness of CM.

**Abstract:**

Background: The incidence of invasive cutaneous melanoma (CM) is increasing in Sweden. The aim was to present age- and sex-specific trends of the age-standardised incidence and the average annual percentage change (AAPC) for in situ and invasive CM. Methods: Joinpoint regression models were used to analyse data from the Swedish Cancer Register and the Swedish Melanoma Registry 1997–2018 (*N* = 35,350 in situ CM; 59,932 CM). Results: The AAPC of CM for women was 4.5 (4.1–5.0; *p* < 0.001) for the period 1997–2018. For men, the APCC was 4.2 (3.0–5.4; *p* < 0.001), with a significantly higher annual percentage change (APC) for the period 2000–2018 (5.0; 4.6–5.4; *p* < 0.001) compared to 1997–1999. An increasing annual incidence of CM ≤ 0.6 mm and 0.7 mm Breslow tumour thickness was found for men with a significant incidence shift for the period 2006–2015, respectively. Similarly for women, with a significantly higher APC for CM ≤ 0.6 mm from 2005. The incidence of intermediate thick CM (2.1–4.0 mm) has not increased since 2011. The incidence of CM > 4.0 mm has been increasing among both sexes, with a significantly lower APC among women from 2005. Conclusions: The incidence of in situ and low-risk CM ≤ 1.0 mm in tumour thickness has been rising among both sexes since the 2000s.

## 1. Introduction

The incidence of invasive cutaneous melanoma (CM) is increasing in Sweden as in the majority of the Western countries with fair-skinned populations, e.g., North America, Northern Europe, Australia and New Zealand [1,2]. The highest incidence rates world-wide have been reported from Australia and New Zealand [3,4]. In Australia, the age-standardised incidence rates in 2018 were 40.4/100,000 cases per year for men and 27.5/100,000 cases per year for women [1]. In all European countries, the incidence rates of CM have increased since the 1950s, with an estimated annual increase of 3–7% over the past decades [5,6]. The incidence rates within Europe vary between countries, with the highest incidence rates found in the western parts of Northern Europe, with incidence rates of 15.0–29.6/100,000 per year [1,5,6,7]. Sweden, Norway and Denmark are the countries with the highest incidence for women in Europe, ranging from 26.2 to 33.1/100,000 per year; these rates are now in the same order of magnitude as those for women in Australia and New Zealand. The incidence rates for men in the same countries are not far behind, with incidence rates of 22.4 to 29.0/100,000 per year. The CM incidence is also increasing in the US population, with an age-standardised incidence rate of 14.9/100,000 per year for men and 11.0/100,000 per year for women [1].

Ultraviolet (UV) radiation is the main risk factor for CM, and an increased UV exposure has mainly contributed to the observed increase in CM incidence world-wide [8,9,10,11]. Different population-based prevention approaches have therefore been implemented to reduce skin cancer incidence. In Australia, the public education to increase the awareness of early CM began in Queensland as early as the 1960s, and these efforts were eventually expanded to include primary prevention through sun protection [10,12,13,14]. In Australia, a recent stabilisation in invasive CM incidence has been observed, which is probably attributed to a decline in younger birth cohorts (4). However, in Europe, no declining incidence shift has been observed [7,15,16,17,18]. Instead, the increase in incidence of both in situ and invasive CM is a considerable burden to public health, and the annual costs of CM management within health care are substantial [19,20,21]. These challenges of CM prevention and care emphasise the continued need for population-based epidemiologic surveillance.

In this report, we present age- and sex-specific trends of the age-standardised incidence and the average annual percentage change (AAPC) for in situ and invasive CM between 1997 and 2018 by using population-based registries: the Swedish Cancer Register (SCR) for in situ CM, and the Swedish Melanoma Registry (SweMR) for information on invasive CM.

## 2. Results

In total, 35,350 in situ CM were recorded between 1997 and 2018 in Sweden: 18,110 (51.2%) cases among women and 17,240 cases (48.8%) among men (Table 1).

The median age at diagnosis for in situ CM was 67 years (interquartile range (IQR): 55–76 years). This corresponded to 65 years for women and 69 years for men. The total number of cases for invasive CM registered between 1997 and 2018 was 59,932 (Table 1). Of these, the number of women and men were comparable (49.2% and 50.8%, respectively). The median age was 65 years at diagnosis (IQR 51–75 years). The median age for women was 62 years, and 67 years for men. The majority of the cases (*n* = 21,352; 35.6%) were T1a CM. T1b corresponded to 9450 (15.8%) cases. The overall median tumour thickness was 0.9 mm (IQR: 0.5–2.0 mm). The median tumour thickness for women was 0.8 mm (IQR: 0.5–1.7 mm), and 1.0 mm (IQR: 0.5–2.2 mm) for men.

### 2.1. Annual Age-Standardised Incidence, 1997–2018

**Figure 1:**
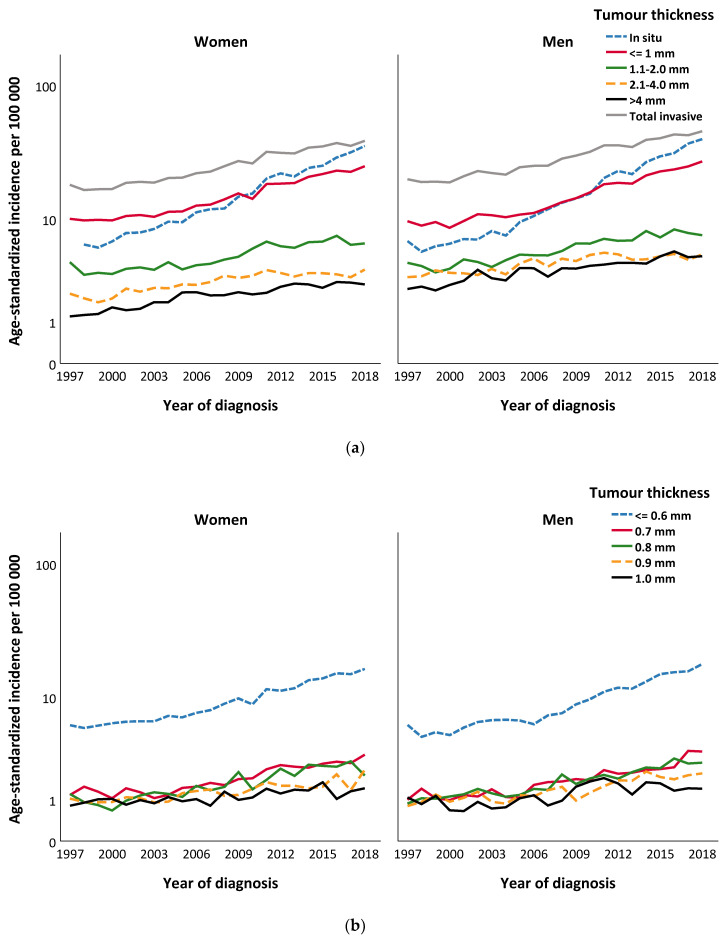

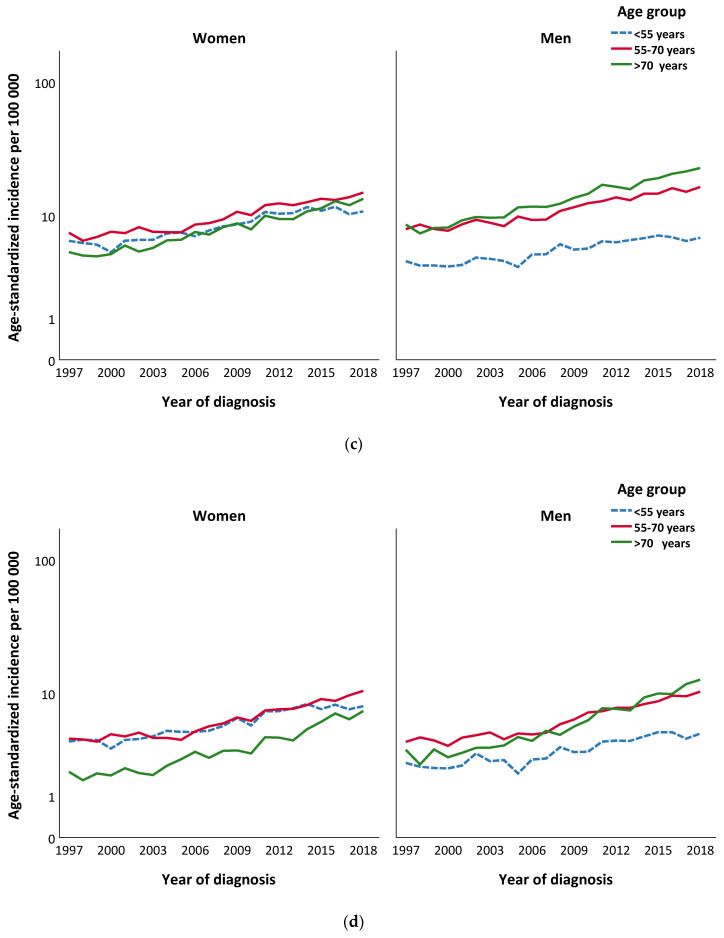

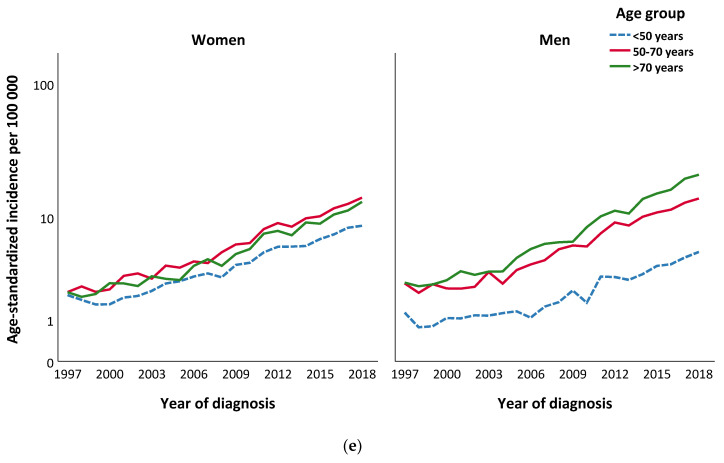
Trends of age-standardised incidence (logarithmic scale) per 100,000 individuals of cutaneous melanomas (CM) among men and women diagnosed in Sweden, 1997–2018, according to: (**a**) T-stage; (**b**) tumour thickness for T1 cutaneous melanoma; (**c**) age; (**d**) age for T1 cutaneous melanoma; and (**e**) age for in situ cutaneous melanoma.

Figure 1 describes the trends of age-standardised incidence (logarithmic scale) of invasive and in situ CM per 100,000 individuals among women and men, 1997–2018, according to tumour thickness and age, as well as for subgroups of T1 CM and in situ CM.

#### 2.1.1. In Situ Cutaneous Melanomas

Between the years 1997 and 2018, the average annual age-standardised incidence of in situ CM was 16.5/100,000 per year which corresponds to 16.2/100,000 per year among women and 17.5/100,000 per year among men (standardised by the Swedish population in the year 2000) (Figure 1a).

#### 2.1.2. Invasive Cutaneous Melanomas

For all patients, the average annual age-standardised incidence of invasive CM was 28.0/100,000 per year between 1997 and 2018, which corresponds to 26.5/100,000 per year among women and 30.7/100,000 per year among men (standardised by the Swedish population in the year 2000). In particular, the increase in CM incidence is higher in women >70 years compared to women <50 years (Figure 1).

### 2.2. Incidence Trends of In Situ and Invasive Cutaneous Melanomas by Tumour Thickness

#### 2.2.1. In Situ Cutaneous Melanomas

Among men, in situ CM significantly increased during the whole study period, but was more prominent (an average annual percentage (APC) 2 of 12.8, 95% CI 11.9–13.7, *p* < 0.001) from the year 2002 (Table 2a).

#### 2.2.2. Invasive Cutaneous Melanomas

The modelled data showed that the overall sex-specific annual age-standardised incidence of invasive CM fluctuated depending on the thickness group. The AAPC of the incidence of invasive CM for women was 4.5 (4.1–5.0; *p* < 0.001) for the total study period, 1997–2018 (Table 2a). For men, the APCC was 4.2 (3.0–5.4; *p* < 0.001), with a significantly higher annual percentage change for the period 2000–2018 (APC2) (5.0; 4.6–5.4; *p* < 0.001) compared to 1997–1999 (APC1) (Table 2a). An increasing annual incidence of CM with tumour thicknesses ≤0.6 mm and 0.7 mm was found for men, with a significant shift of incidence from 2006 and 2015, respectively (Table 2a, Figure 1b). A similar trend was shown for women, where CM with a thickness of ≤0.6 mm increased for the entire study period, but with a significantly higher APC from 2005. The incidence of 0.7 mm thick CM decreased between 1997 and 2003, but with a significant shift of an increasing incidence from 2003 in females (Table 2a, Figure 1b).

For both female and male patients, the incidence of CM with an intermediate thickness of 2.1–4.0 mm has not increased since 2011 (Table 2a; Figure 1a). The incidence of CM > 4.0 mm increased among both sexes during the study period, but with a significant trend of a lower APC among women from the year 2005 (Table 2a, Figure S1).

### 2.3. Incidence Trends of Invasive Cutaneous Melanomas by Age

In both women and men, the AAPC of the incidence of invasive CM based on age group significantly increased between 1997 and 2018 for all patients (AAPC_women<50 years_ 3.3, 1.9–4.8, *p* < 0.001; AAPC_men<50 years_ 3.1, 2.5–3.6, *p* < 0.001; AAPC_women 50–70 years_ 4.8, 3.6–6.0, *p* < 0.001; AAPC_men 50–70 years_ 5.8, 4.7–6.9, *p* < 0.001; AAPC_women>70 years_ 5.5, 5.0–6.0, *p* < 0.001; AAPC_men>70 years_ 5.7, 5.3–6.1, *p* < 0.001) (Table 2a–d).

Interestingly, the APC for subgroups of CM ≤ 0.6 mm significantly increased among men older than 70 years (APC2_CM≤0.6 mm from year 2001_ 10.2, 9.1–11.4, *p* < 0.001; APC2_CM 0.7 mm from year 2015_ 23.2, 7.9–40.7, *p* = 0.004) (Table 2d). A trend of decreasing incidence of T4 CM among women aged 50–70 was noted from the year 2010 (−7.6; −16.4–1.9; *p* = 0.108), but the incidence of T4 CM still increased in the age group >70 years, although with a significantly lower incidence rate.

## 3. Discussion

By using the population-based, high-coverage SweMR, we have pointed out specific age- and sex-specific trends in CM incidence in the Swedish population which may contribute to the implementation of new CM prevention strategies. The incidence of in situ and low-risk invasive CM ≤ 1.0 mm in tumour thickness has been rising among both sexes since the 2000s. Interestingly, this has appeared later among males compared to females, with a trend of an increased incidence of thinner CM towards men > 70 years. CM > 4.0 mm in tumour thickness with a high risk of recurrences and CM-related death still continue to increase among both sexes, but with a slightly lower increase among females. The results may indicate sex-specific differences of CM awareness and health care-seeking behaviour.

We show that the annual age-standardised incidence of both in situ and invasive CM continues to increase among both men and women in Sweden, which is in line with the incidence trends in almost all Caucasian populations world-wide [1,2,5,22,23]. For example, the highest age-standardised CM incidence rates within Europe for 2018 ranged from 15.3 to 33.1/100,000 per year [1,5,6,15]. Behavioural changes in lifestyle factors associated with an increased exposure to UV radiation during the decades from 1950 and later have probably contributed to the increase in CM incidence [5,8,9]. The sun protection campaigns have generally been less coordinated in Europe and in the US compared to the early national efforts in Australia and New Zealand [4,14,24]. The trend of increasing CM incidence world-wide evokes the question of whether there are additional risk factors other than UV radiation. An association between commonly used drugs and risk for CM has been reported [25,26,27]. Some medications have secondary immuno-modulating effects in mouse models and human cell lines, but the clinical impact has to be validated [28,29,30,31]. However, the incidence rates seem to stabilise or decrease among younger individuals in New Zealand and Australia, as well as in some parts of Europe, Canada and the US [3,4,5,32,33]. We found a similar trend for men aged below 50 years in Sweden. A possible explanation may be reduced sun exposure in younger individuals due to sun protection information, less outdoor work and reduced outdoor leisure activities [14,34,35].

Ongoing trends towards thinner (≤1.0 mm in tumour thickness) CM have been described in several countries with fair-skinned populations [3,16,17,33,36]. The increase in the annual incidence of in situ CM seems to follow the same pattern as for invasive tumours. Some of these reports have even shown that the annual incidence rate of in situ CM has been increasing more rapidly than that of invasive CM in Queensland, Australia; Denmark, the Netherlands; and the US [3,17,33].

Moreover, the increase in CM incidence in Sweden corresponds to the CM incidence among women in Australia, but the increase corresponds to thin low-risk CM with a good prognosis [1,37]. However, we also found that thick CM > 4.0 mm are still increasing among men and women, which shows that the increase in age-standardised CM incidence in Sweden is not only attributed to improved detection of low-risk tumours.

In a recent publication by H. Gilbert Welch et al. with the title The Rapid Rise in Cutaneous Melanoma Diagnoses [38], the authors pointed out that considerable disagreement still exists as to whether this increased incidence represents a true epidemic, or rather, an “epidemic of diagnosis”. The authors stated that “The most important step to break the cycle of melanoma overdiagnosis is to stop population-wide screening for skin cancer”. In Sweden, melanomas are mostly diagnosed in primary care by general practitioners. For dermatologic specialist care, a referral is mandatory, and screening is not reimbursed in the tax funded Swedish health care system. Screening for melanoma is basically absent in Sweden, besides targeted screening in high risk persons, e.g., members of melanoma-prone families and patients with a history of multiple melanomas. The recent trend of an increasing proportion of pre-invasive and early invasive CM could be explained by the more widespread use of dermatoscopy since the early 2000s in Sweden [39]. Dermatoscopy, with better diagnostic accuracy for CM than naked eye assessment, could have a population-wide impact by improving early detection and diagnosing thinner CM. The features of early melanomas can be very subtle clinically, therefore escaping detection until dermatoscopy is applied [40,41]. Pre-invasive and early invasive CM also tend to be histopathologically ambiguous and therefore more prone to both over and underdiagnosis, to an extent that is still not settled [42,43].

The main strengths of the present study include prospectively collected population-based data from the high-coverage, national SweMR, with detailed clinico-pathological information on all invasive CM diagnosed in Sweden, making the results generalizable to other populations. The weaknesses include less detailed clinical information on in situ CM.

In conclusion, although we show an alarming increase in the annual age-standardised incidence of in situ and invasive CM in Sweden, we found that this largely corresponds to a trend of in situ and low-risk CM ≤ 1.0 mm in tumour thickness in both sexes since the 2000s.

## 4. Patients and Methods

The nationwide, population-based SweMR includes prospectively collected clinico-pathological data on invasive CM in Sweden since 1990. The register was initiated by the multidisciplinary Swedish Melanoma Study Group, and has had a high coverage (99%) since 1996, as compared to the reference register, the SCR [44,45]. The SCR is maintained by the National Board of Health and Welfare, and reporting to this register is mandatory both for clinicians and for the diagnosing pathologists. The SCR includes invasive as well as in situ CM.

Initially, reporting to the SweMR was lower in some parts of Sweden between 1990 and 1996 [44]. This study therefore includes CM patients diagnosed from 1997 and onwards, to obtain high coverage national data. Between 1997 and 2018, 59,932 invasive CM were recorded in the SweMR. The corresponding number of in situ CM was 35,350 cases.

Data on in situ (Tis) CM were obtained from the SCR and included sex and age (<50, 50–70, >70 years).

The patients were classified according to the 8th edition of the American Joint Committee on Cancer (AJCC) 8 staging for invasive CM [46]. Information on clinico-pathological variables was assessed from the SweMR and included: sex, age (<50, 50–70, >70 years), Breslow tumour thickness (continuous), ulceration status (absent, present, missing information), histologic subtype of CM (superficial spreading (SSM), nodular (NM), lentigo malignant melanoma (LMM), acral lentiginous melanoma (ALM), other, missing information) and T-stage (TX, T1, T1a-b, T2, T2a-b, T3, T3a-b, T4, T4a-b). This corresponds to T-stage according to AJCC 8: TX: primary CM thickness cannot be assessed; T1: ≤1.0 mm, ulceration unknown or unspecified and not classified as T1a or T1b; T1a: <0.8 mm without ulceration; T1b: <0.8 mm with ulceration and 0.8–1.0 mm with/without ulceration; T2: 1.1–2.0 mm, ulceration unknown or unspecified; T2a: 1.1–2.0 mm without ulceration; T2b: 1.1–2.0 mm with ulceration; T3: 2.1–4.0 mm, ulceration unknown or unspecified; T3a: 2.1–4.0 mm without ulceration; T3b: 2.1–4.0mm with ulceration; T4: >4.0 mm, ulceration unknown or unspecified; T4a: >4.0 mm without ulceration; T4b: >4.0 mm with ulceration.

### Statistical Analysis

The statistical analysis was performed for men and women separately. All incidence rates per 100,000 inhabitants were calculated for each year of diagnosis and standardised by the Swedish population in the year 2000. Joinpoint regression models were used to evaluate whether temporal trends had statistically significant points of change, and to estimate the average annual percentage change (AAPC) with subdivisions of sex, age-groups and tumour thickness [47]. The time periods (tp) were autogenerated by the joinpoint analyses, and for each time period, the annual percentage change (APC(tp)) was calculated. For this purpose, we used the Joinpoint Regression Software developed by the Surveillance Research Program of the US National Cancer Institute [48,49]. The Joinpoint Regression Program used a permutation test to find the optimal number of joinpoints. The Program ran 4499 permutations to select the model with an overall significance level of 0.05. The analyses allowed a maximum of one joinpoint, and the maximum number of estimated APCs was consequently two. The statistical analyses were performed using SPSS version 27 software (IBM, Armonk, NY, USA) or R version 4.0.0. (R Core Team, Vienna, Austria). Missing data were handled by pairwise deletion.

## 5. Conclusions

In conclusion, although we show an alarming increase in the annual age-standardised incidence of in situ and invasive CM in Sweden, we found that this largely corresponds to a trend of in situ and low-risk CM ≤ 1.0 mm in tumour thickness in both sexes since the 2000s. These trends may be a result of more effective secondary prevention efforts, including an improved understanding and awareness of the disease both among clinicians and the public. Still, the incidence of high-risk CM continues to rise among both sexes. Prevention efforts such as coordinated and repeated national primary prevention campaigns are needed, including an improved understanding of putative novel risk factors and biomarkers in high-risk populations, and targeted towards specific subgroups (e.g., children/young adults) and high-risk individuals (e.g., older men).

## Figures and Tables

**Table 1:** Clinico-pathological characteristics of in situ and invasive cutaneous melanomas (CM) diagnosed in Sweden, 1997–2018.

Clinico-Pathological Characteristics	All CM N (%)	CM in Women N (%)	CM in Men N (%)
In situ CM	35,350	18,110 (51.2)	17,240 (48.8)
Age (median IQR)	67.0 (55.0; 76.0)	65.0 (52.0; 75.0)	69.0 (59.0; 77.0)
Age group			
<50 years	6585 (18.6)	4220 (23.3)	2365 (13.7)
50–70 years	14,413 (40.8)	7200 (39.8)	7213 (41.8)
>70 years	14,352 (40.6)	6690 (36.9%)	7662 (44.4)
Invasive CM	59,932	29,503 (49.2)	30,429 (50.8)
Age (median IQR)	65.0 (51.0; 75.0)	62.0 (48.0; 75.0)	67.0 (55.0; 76.0)
Age group (years)			
<50	14,100 (23.5)	8607 (29.2)	5493 (18.1)
50–70	23,866 (39.8)	11,104 (37.6)	12,762 (41.9)
>70	21,965 (36.7)	9792 (33.2)	12,173 (40.0)
Breslow tumour thickness (mm; median IQR)	0.90 (0.50; 2.00)	0.8(0.5; 1.70)	1.00 (0.5; 2.2)
Ulceration status			
Absent	42,895 (71.6)	21,471 (72.8)	21,424 (70.4)
Present	10,702 (17.9)	4749 (16.1)	5953 (19.6)
Missing	6335 (10.6)	3283 (11.1)	3052 (10.0)
Histopathologic subtype			
SSM	36,649 (61.2)	18,445 (62.5)	18,204 (59.8)
NM	4100 (6.8)	2024 (6.86)	2076 (6.82)
LM	9548 (15.9)	4194 (14.2)	5354 (17.6)
ALM	667 (1.1)	402 (1.4)	265 (0.9)
Other	7281 (12.1)	3590 (12.2)	3691 (12.1)
Missing	1687 (2.8)		
T-stage			
TX	2015 (3.4)	1055 (3.6)	960 (3.2)
T1	2032 (3.4)	1117 (3.8)	915 (3.0)
T1a	21,352 (35.6)	11,118 (37.7)	10,234 (33.6)
T1b	9450 (15.8)	4755 (16.1)	4695 (15.4)
T2	1046 (1.8)	540 (1.8)	506 (1.7)
T2a	8298 (13.8)	4017 (13.6)	4281 (14.1)
T2b	2046 (3.4)	990 (3.4)	1056 (3.5)
T3	622 (1.0)	281 (1.0)	341 (1.4)
T3a	3674 (6.1)	1699 (5.8)	1975 (6.5)
T3b	3260 (5.4)	1365 (4.6)	1895 (6.2)
T4	408 (0.68)	167 (0.6)	241 (0.8)
T4a	1796 (3.0)	743 (2.5)	1053 (3.5)
T4b	3933 (6.6)	1656 (5.6)	2277 (7.5)

CM: Cutaneous Melanoma. IQR: Interquartile range. T-stage according to AJCC 8: TX: Primary CM thickness cannot be assessed; T1: ≤1.0 mm, ulceration unknown or unspecified and not classified as T1a or T1b; T1a: <0.8 mm without ulceration; T1b: <0.8 mm with ulceration and 0.8–1.0 mm with/without ulceration; T2: 1.1–2.0 mm, ulceration unknown or unspecified; T2a: 1.1–2.0 mm without ulceration; T2b: 1.1–2.0 mm with ulceration; T3: 2.1–4.0 mm, ulceration unknown or unspecified; T3a: 2.1–4.0 mm without ulceration; T3b: 2.1–4.0 mm with ulceration; T4: >4.0 mm, ulceration unknown or unspecified; T4a: >4.0 mm without ulceration; T4b: >4.0 mm with ulceration. SSM: superficial spreading melanoma; NM: nodular melanoma; LMM: lentigo malignant melanoma; ALM: acral lentiginous melanoma.

**Table 2:** Annual percentage change (APC) in age-standardised incidence rates for in situ and invasive cutaneous melanoma in Sweden, 1997–2018, by: (**a**) sex and tumour thickness for all ages; (**b**) sex and tumour thickness for age group <50 years (**c**) sex and tumour thickness for age group 50–70 years; (**d**). sex and tumour thickness for age group >70 years.

(a)													
All Ages	Women												
	1997–2018				Period 1				Y	Period 2			
	AAPC	LCL	UCL	*p*-Value	APC1	LCL	UCL	*p*-Value		APC2	UCL	LCL	*p*-Value
**Tumour Thickness**													
**All**	4.5	4.1	5.0	<0.001		-	-	-	-	-	-	-	-
**≤0.6**	5.4	4.5	6.3	<0.001	2.9	0.8	5.1	0.009	2005	7.0	6.2	7.7	<0.001
**0.7**	4.4	2.6	6.2	<0.001	−1.0	−6.6	5.0	0.724	2003	6.6	5.4	7.8	<0.001
**0.8**	5.7	4.3	7.0	<0.001	-	-	-	-	-	-	-	-	-
**0.9**	3.8	2.7	4.8	<0.001	-	-	-	-	-	-	-	-	-
**1.0**	2.5	1.5	3.5	<0.001	-	-	-	-	-	-	-	-	-
**1.1–2.0**	3.6	2.8	4.4	<0.001	-	-	-	-	-	-	-	-	-
**2.1–4.0**	2.9	1.9	3.9	<0.001	4.5	3.4	5.5	<0.001	2011	−0.1	−2.4	2.2	0.904
**>4**	4.6	3.5	5.6	<0.001	7.7	5.1	10.4	<0.001	2005	2.6	1.8	3.5	<0.001
**Missing**	2.5	−0.0	5.1	0.053	8.4	4.8	12.1	<0.001	2009	−4.9	−8.9	−0.6	0.027
**In situ**	10.0	9.4	10.5	<0.001	-	-	-	-	-	-	-	-	-
**All Ages**	**Men**												
	**1997–2018**				**Period 1**				**Y**	**Period 2**			
	**AAPC**	**LCL**	**UCL**	***p*-Value**	**APC1**	**LCL**	**UCL**	***p*-Value**		**APC2**	**LCL**	**UCL**	***p*-Value**
**Tumour Thickness**													
**All**	4.2	3.0	5.4	<0.001	−0.4	−8.4	8.3	0.920	2000	5.0	4.6	5.4	<0.001
**≤0.6**	6.3	5.2	7.4	<0.001	3.2	0.9	5.5	0.009	2006	8.7	7.7	9.8	<0.001
**0.7**	6.4	4.2	8.7	<0.001	5.0	3.7	6.4	<0.001	2015	15.2	0.4	32.2	0.044
**0.8**	5.6	4.8	6.4	<0.001	-	-	-	-	-	-	-	-	-
**0.9**	4.4	3.4	5.5	<0.001	-	-	-	-	-	-	-	-	-
**1.0**	3.3	1.6	5.0	<0.001	-	-	-	-	-	-	-	-	-
**1.1–2.0**	3.9	3.3	4.5	<0.001	-	-	-	-	-	-	-	-	-
**2.1–4.0**	2.1	1.1	3.2	<0.001	3.4	2.3	4.5	<0.001	2011	−0.4	−2.8	2.1	0.753
**>4**	3.4	2.8	4.0	<0.001		-	-	-	-	-	-	-	-
**Missing**	2.3	−0.6	5.3	0.119	6.7	2.7	11.0	0.003	2009	−3.3	−8.0	1.6	0.168
**In situ**	10.2	8.8	11.6	<0.001	2.4	−2.6	7.7	0.334	2002	12.8	11.9	13.7	<0.001
**(b)**													
**Age Group: <50**	**Women**												
	**1997–2018**				**Period 1**				**Y**	**Period 2**			
	**AAPC**	**LCL**	**UCL**	***p*-Value**	**APC1**	**LCL**	**UCL**	***p*-Value**		**AAPC**	**LCL**	**UCL**	***p*-Value**
**Tumour Thickness**													
**All**	3.3	1.9	4.8	<0.001	4.4	3.6	5.2	<0.001	2015	–2.9	–11.	6.7	0.513
**≤0.6**	4.8	4.1	5.4	<0.001	-	-	-	-	-	-	-	-	-
**0.7**	3.7	2.3	5.2	<0.001	-	-	-	-	-	-	-	-	-
**0.8**	1.5	–5.6	9.2	0.680	5.5	3.0	8.1	<0.001	2016	–29.4	–68.1	55.	0.366
**0.9**	3.8	2.1	5.5	<0.001	-	-	-	-	-	-	-	-	-
**1.0**	1.1	–0.8	3.1	0.247	-	-	-	-	-	-	-	-	-
**1.1–2.0**	2.8	1.4	4.2	<0.001	-	-	-	-	-	-	-	-	-
**2.1–4.0**	3.7	2.5	4.9	<0.001	-	-	-	-	-	-	-	-	-
**>4**	2.2	–0.1	4.7	0.061	-	-	-	-	-	-	-	-	-
**Missing**	1.4	–3.2	6.4	0.552	7.6	3.1	12.	0.002	2012	–12.6	–24.2	0.8	0.063
**Age Group: <50**	**Men**												
	**1997–2018**				**Period 1**				**Y**	**Period 2**			
	**AAPC**	**LCL**	**UCL**	***p*-Value**	**APC1**	**LCL**	**UCL**	***p*-Value**		**AAPC**	**LCL**	**UCL**	***p*-Value**
**Tumour Thickness**													
**All**	3.1	2.5	3.6	<0.001	-	-	-	-	-	-	-	-	-
**≤0.6**	4.4	3.3	5.6	<0.001	-	-	-	-	-	-	-	-	-
**0.7**	4.1	2.4	5.8	<0.001	-	-	-	-	-	-	-	-	-
**0.8**	2.9	1.2	4.7	0.002	-	-	-	-	-	-	-	-	-
**0.9**	2.5	0.9	4.0	0.002	-	-	-	-	-	-	-	-	-
**1.0**	2.4	0.8	4.0	0.005	-	-	-	-	-	-	-	-	-
**1.1–2.0**	2.3	1.1	3.5	0.001	-	-	-	-	-	-	-	-	-
**2.1–4.0**	0.8	–0.4	2.1	0.200	-	-	-	-	-	-	-	-	-
**>4**	0.1	–1.6	1.9	0.886	-	-	-	-	-	-	-	-	-
**Missing**	0.9	–4.73	6.9	0.749	10.6	1.9	20.2	0.019	2008	–8.76	–16.9	0.2	0.055
**(c)**													
**Age Group: 50–70**	**Women**												
	**1997–2018**				**Period 1**				**Y**	**Period 2**			
	**AAPC**	**LCL**	**UCL**	***p*-Value**	**APC1**	**LCL**	**UCL**	***p*-Value**		**AAPC**	**LCL**	**UCL**	***p*-Value**
**Tumour Thickness**													
**All**	4.8	3.6	6.0	<0.001	1.3	−1.5	4.1	0.344	2005	7.0	6.0	8.1	<0.001
**≤0.6**	5.1	3.5	6.7	<0.001	-	-	-	-	-	-	-	-	-
**0.7**	5.7	4.2	7.3	<0.001	-	-	-	-	-	-	-	-	-
**0.8**	2.6	1.5	3.8	<0.001	-	-	-	-	-	-	-	-	-
**0.9**	2.6	1.3	3.9	<0.001	-	-	-	-	-	-	-	-	-
**1.0**	3.6	2.7	4.6	<0.001	-	-	-	-	-	-	-	-	-
**1.1–2.0**	2.5	1.3	3.7	<0.001	-	-	-	-	-	-	-	-	-
**2.1–4.0**	2.7	1.4	4.1	<0.001	-	-	-	-	-	-	-	-	-
**>4**	1.8	−2.9	6.9	0.449	8.2	2.2	14.	0.010	2010	−7.6	−16.4	1.9	0.108
**Missing**	3.9	2.9	5.0	<0.001	2.0	−0.7	4.9	0.144	2004	4.9	4.1	5.7	<0.001
**Age Group: 50–70**	**Men**												
	**1997–2018**				**Period 1**				**Y**	**Period 2**			
	**AAPC**	**LCL**	**UCL**	***p*-Value**	**APC1**	**LCL**	**UCL**	***p*-Value**		**AAPC**	**LCL**	**UCL**	***p*-Value**
**Tumour Thickness**													
**All**	5.8	4.7	6.9	<0.001	3.7	1.4	6.1	0.003	2006	7.4	6.3	8.5	<0.001
**≤0.6**	4.4	2.1	6.7	<0.001	−0.3	−5.6	5.2	0.909	2005	7.4	5.3	9.4	0.000
**0.7**	4.8	3.6	6.0	<0.001	-	-	-	-	-	-	-	-	-
**0.8**	3.4	2.0	4.8	<0.001	-	-	-	-	-	-	-	-	-
**0.9**	2.2	0.3	4.1	0.023	-	-	-	-	-	-	-	-	-
**1.0**	3.6	2.9	4.3	<0.001	-	-	-	-	-	-	-	-	-
**1.1–2.0**	1.8	0.9	2.7	<0.001	-	-	-	-	-	-	-	-	-
**2.1–4.0**	2.2	1.2	3.2	<0.001	-	-	-	-	-	-	-	-	-
**>4**	1.4	−1.16	4.0	0.267	-	-	-	-	-	-	-	-	-
**Missing**	4.1	3.7	4.6	<0.001	-	-	-	-	-	-	-	-	-
**(d)**													
**Age Group: >70**	**Women**												
	**1997–2018**				**Period 1**				**Y**	**Period 2**			
	**AAPC**	**LCL**	**UCL**	***p*-Value**	**APC1**	**LCL**	**UCL**	***p*-Value**		**AAPC**	**LCL**	**UCL**	***p*-Value**
**Tumour Thickness**													
**All**	5.5	5.0	6.0	<0.001	-	-	-	-	-	-	-	-	-
**≤0.6**	8.2	7.1	9.3	<0.001	-	-	-	-	-	-	-	-	-
**0.7**	7.8	6.0	9.7	<0.001	-	-	-	-	-	-	-	-	-
**0.8**	6.3	1.4	11.	0.010	9.2	7.0	11.5	<0.001	2016	−17.9	−49.8	34.	0.410
**0.9**	5.8	4.1	7.6	<0.001	-	-	-	-	-	-	-	-	-
**1.0**	3.9	2.3	5.5	<0.001	-	-	-	-	-	-	-	-	-
**1.1–2.0**	4.2	3.4	5.1	<0.001	-	-	-	-	-	-	-	-	-
**2.1–4.0**	3.0	2.1	4.0	<0.001	-	-	-	-	-	-	-	-	-
**>4**	5.2	3.8	6.6	<0.001	7.6	4.6	10.	<0.001	2006	3.4	2.1	4.8	<0.001
**Missing**	2.3	0.0	4.7	0.043	-	-	-	-	-	-	-	-	-
**Age Group: >70**	**Men**												
	**1997–2018**				**Period 1**				**Y**	**Period 2**			
	**AAPC**	**LCL**	**UCL**	***p*-Value**	**APC1**	**LCL**	**UCL**	***p*-Value**		**AAPC**	**LCL**	**UCL**	***p*-Value**
**Tumour Thickness**													
**All**	5.7	5.3	6.1	<0.001	-	-	-	-	-	-	-	-	-
**≤0.6**	6.9	4.2	9.6	<0.001	−6.1	−18.2	7.6	0.340	2001	10.2	9.1	11.4	<0.001
**0.7**	8.3	6.1	10.6	<0.001	6.0	4.5	7.6	<0.001	2015	23.2	7.9	40.7	0.004
**0.8**	8.0	6.2	9.7	<0.001	-	-	-	-	-	-	-	-	-
**0.9**	6.5	4.6	8.4	<0.001	-	-	-	-	-	-	-	-	-
**1.0**	4.6	1.8	7.4	0.002	-	-	-	-	-	-	-	-	-
**1.1–2.0**	4.8	3.8	5.8	<0.001	-	-	-	-	-	-	-	-	-
**2.1–4.0**	2.7	1.9	3.5	<0.001	-	-	-	-	-	-	-	-	-
**>4**	4.1	3.3	5.0	<0.001	-	-	-	-	-	-	-	-	-
**Missing**	2.5	0.4	4.6	0.018	-	-	-	-	-	-	-	-	-

AAPC: average annual percent change, APC1: annual percent change in the first period, APC2: annual percent change in the second period, Y = starting year for Period 2, LCL: lower confidence limit, UCL: upper confidence limit.

## Data Availability

Data are available from the corresponding authors.

## Supplementary Materials

The following are available online at https://www.mdpi.com/article/10.3390/cancers13112838/s1, Figure S1. Trends in annual percentage change (APC) in age-standardized incidence rates for in situ and invasive cutaneous melanoma by tumour thickness and sex, in Sweden 1997–2018.
