# Supplementary material for: Trend Shifts in Age-Specific Incidence for In Situ and Invasive Cutaneous Melanoma in Sweden
Source: Cancers (Basel). 2021 Jun 7;13(11):2838. doi: 10.3390/cancers13112838 (PMC8201382; doi:10.3390/cancers13112838)
Supplement: Supplementary file 1 [file cancers-13-02838-s001.zip › cancers-1208617-SI.pdf]

Figure S1. Trends in annual percentage change (APC) in age-standardized incidence rates for *in situ* and invasive cutaneous melanoma by tumour thickness and sex, in Sweden 1997-2018.

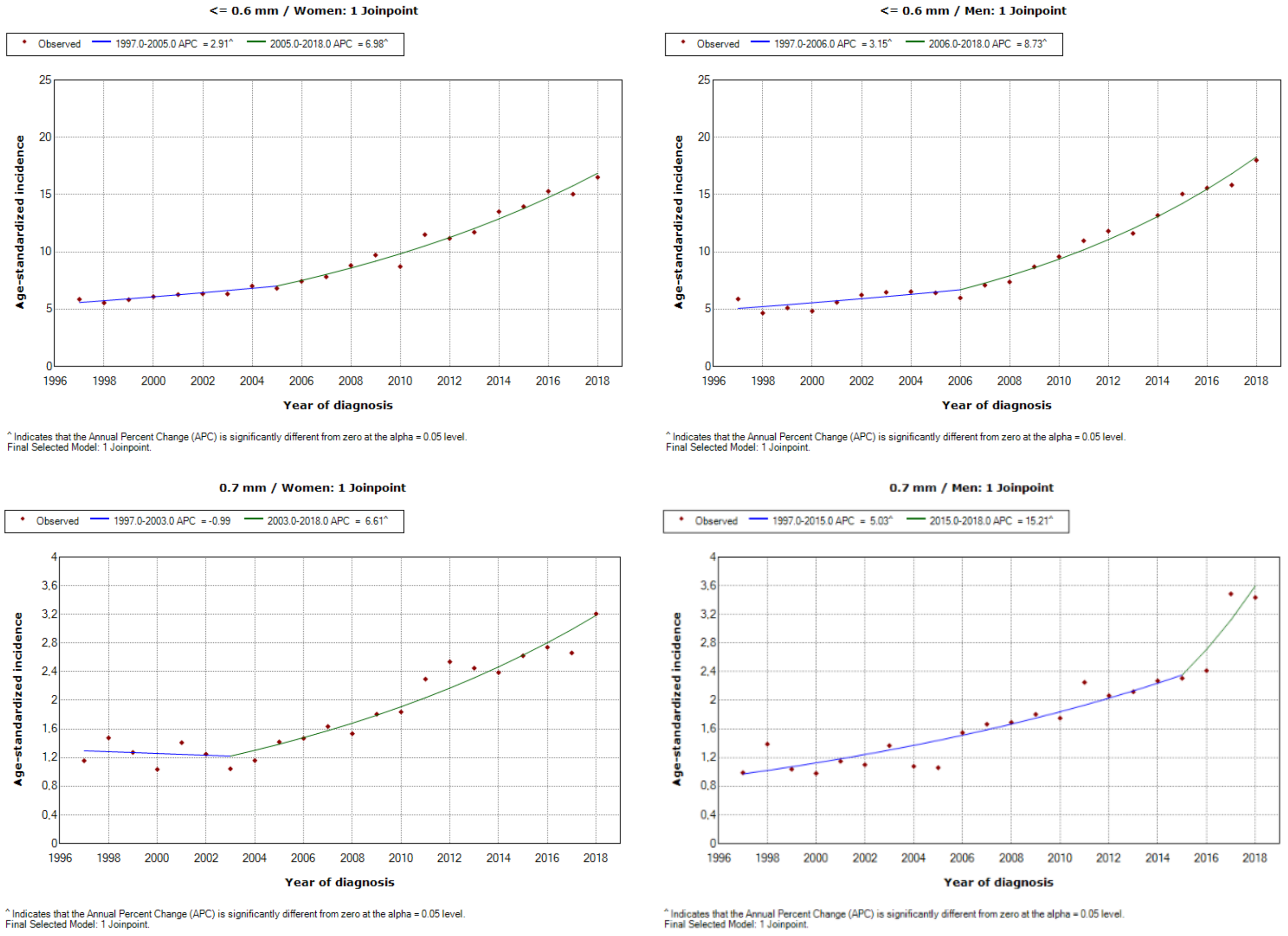

0.8 mm / Women: 0 Joinpoints

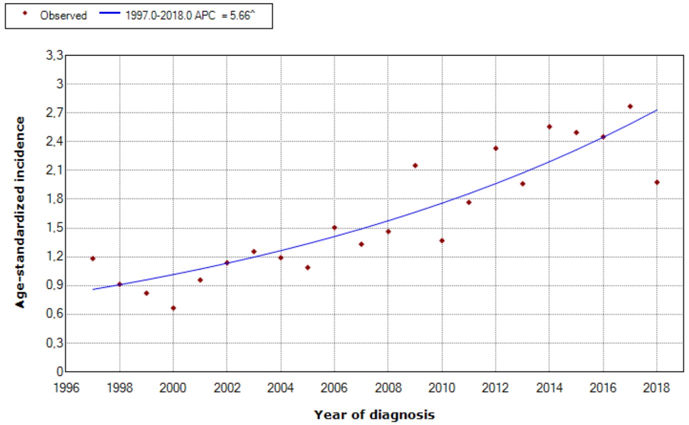

^ Indicates that the Annual Percent Change (APC) is significantly different from zero at the alpha = 0.05 level.
Final Selected Model: 0 Joinpoints.

0.8 mm / Men: 0 Joinpoints

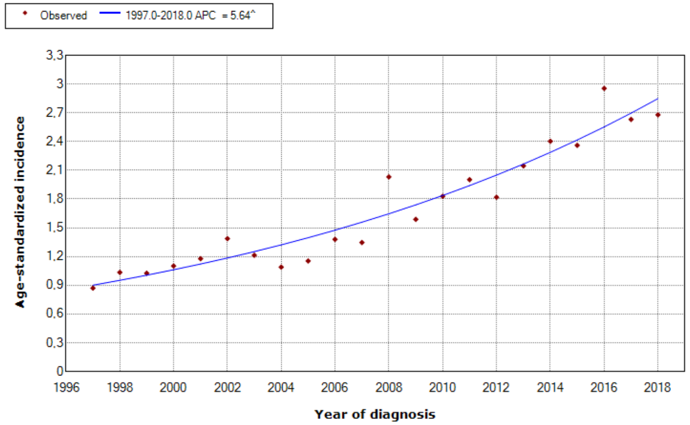

^ Indicates that the Annual Percent Change (APC) is significantly different from zero at the alpha = 0.05 level.
Final Selected Model: 0 Joinpoints.

0.9 mm / Women: 0 Joinpoints

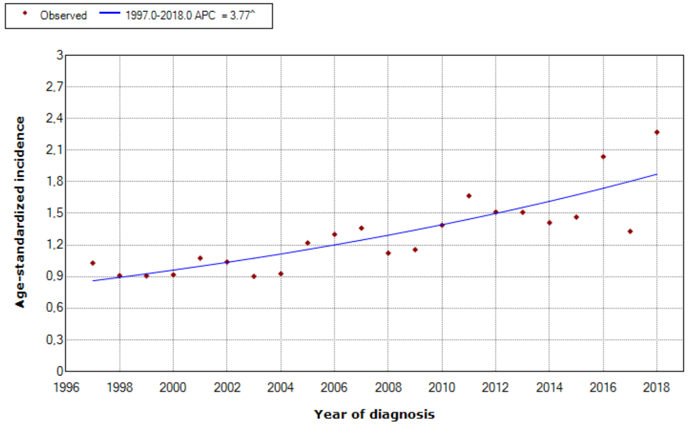

^ Indicates that the Annual Percent Change (APC) is significantly different from zero at the alpha = 0.05 level.
Final Selected Model: 0 Joinpoints.

0.9 mm / Men: 0 Joinpoints

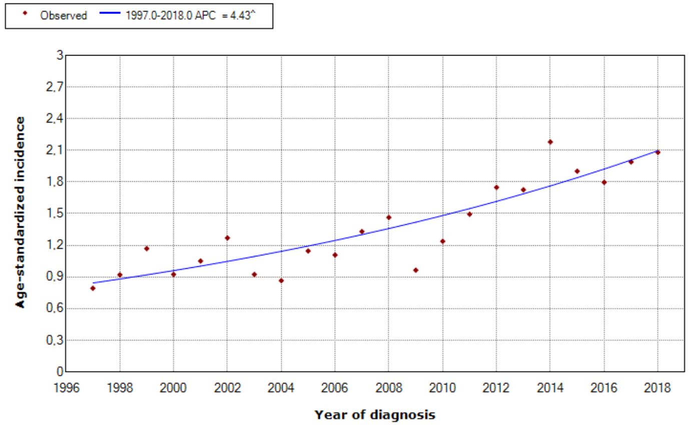

^ Indicates that the Annual Percent Change (APC) is significantly different from zero at the alpha = 0.05 level.
Final Selected Model: 0 Joinpoints.

1.0 mm / Women: 0 Joinpoints

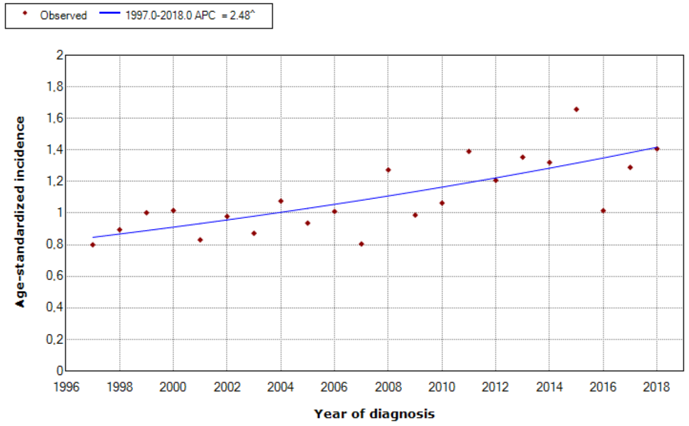

^ Indicates that the Annual Percent Change (APC) is significantly different from zero at the alpha = 0.05 level.
Final Selected Model: 0 Joinpoints.

1.0 mm / Men: 0 Joinpoints

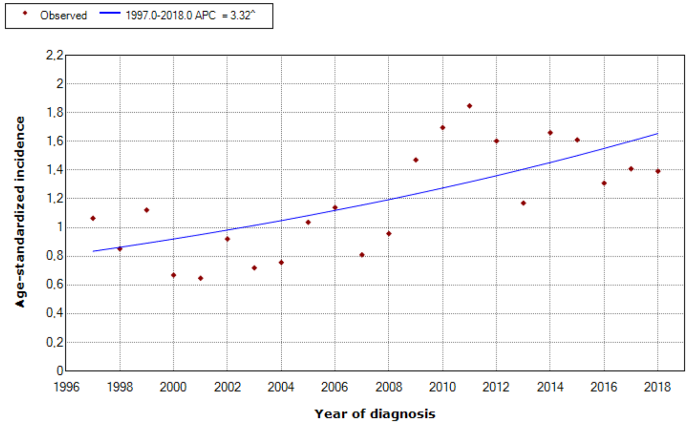

^ Indicates that the Annual Percent Change (APC) is significantly different from zero at the alpha = 0.05 level.
Final Selected Model: 0 Joinpoints.

1.1-2.0 mm / Women: 0 Joinpoints

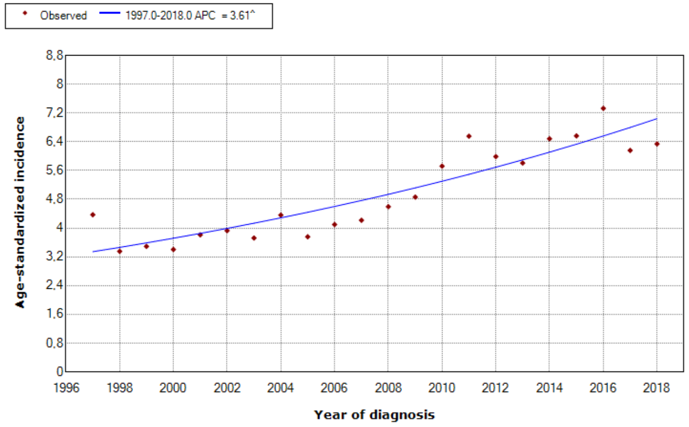

^ Indicates that the Annual Percent Change (APC) is significantly different from zero at the alpha = 0.05 level.
Final Selected Model: 0 Joinpoints.

1.1-2.0 mm / Men: 0 Joinpoints

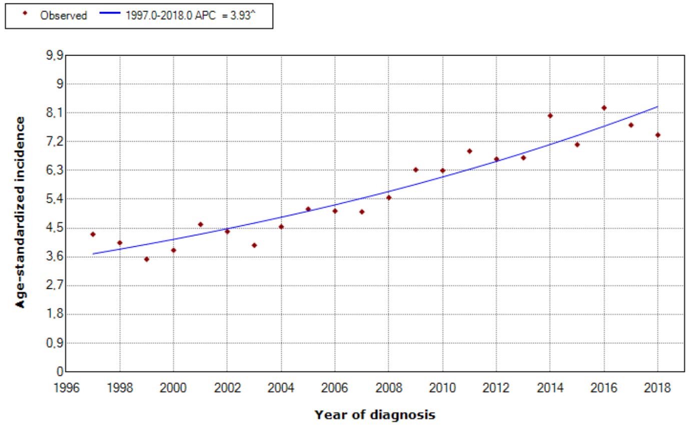

^ Indicates that the Annual Percent Change (APC) is significantly different from zero at the alpha = 0.05 level.
Final Selected Model: 0 Joinpoints.

2.1-4.0 mm / Women: 1 Joinpoint

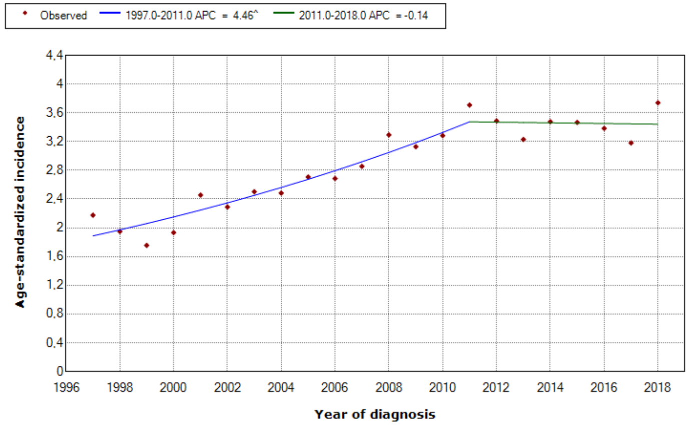

^ Indicates that the Annual Percent Change (APC) is significantly different from zero at the alpha = 0.05 level.
Final Selected Model: 1 Joinpoint.

2.1-4.0 mm / Men: 1 Joinpoint

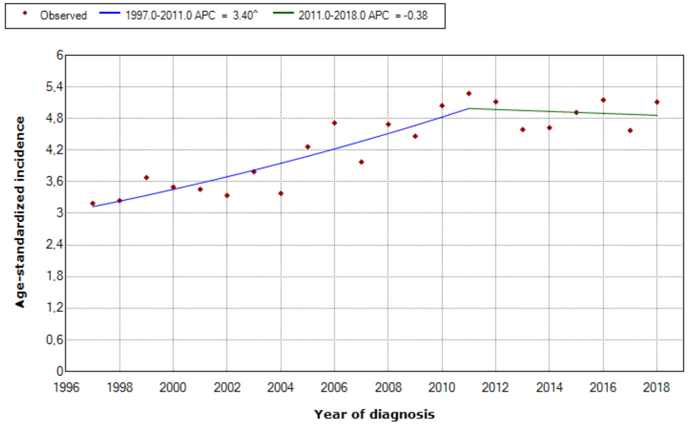

^ Indicates that the Annual Percent Change (APC) is significantly different from zero at the alpha = 0.05 level.
Final Selected Model: 1 Joinpoint.

> 4.0 mm / Women: 1 Joinpoint

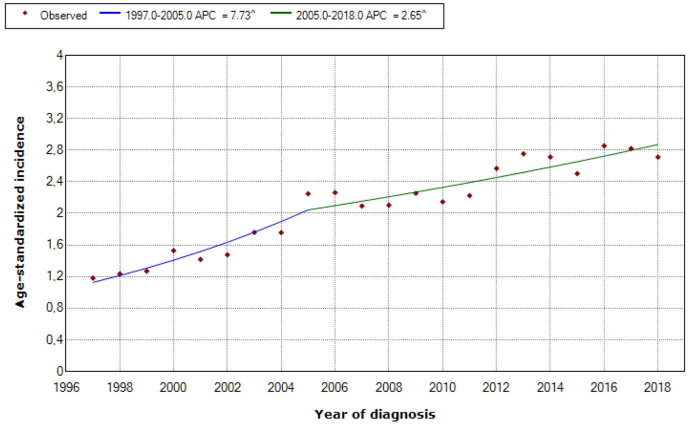

^ Indicates that the Annual Percent Change (APC) is significantly different from zero at the alpha = 0.05 level.
Final Selected Model: 1 Joinpoint.

> 4.0 mm / Men: 0 Joinpoints

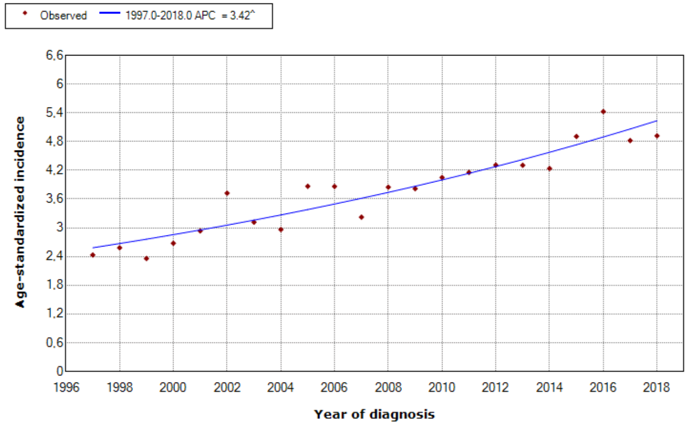

^ Indicates that the Annual Percent Change (APC) is significantly different from zero at the alpha = 0.05 level.
Final Selected Model: 0 Joinpoints.

All / Women: 0 Joinpoints

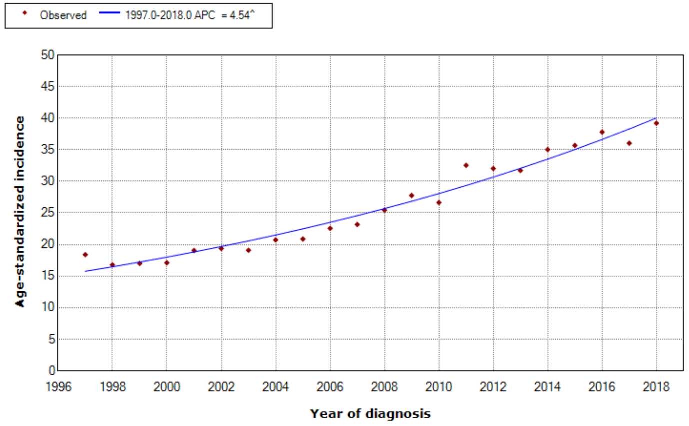

^ Indicates that the Annual Percent Change (APC) is significantly different from zero at the alpha = 0.05 level.
Final Selected Model: 0 Joinpoints.

All / Men: 1 Joinpoint

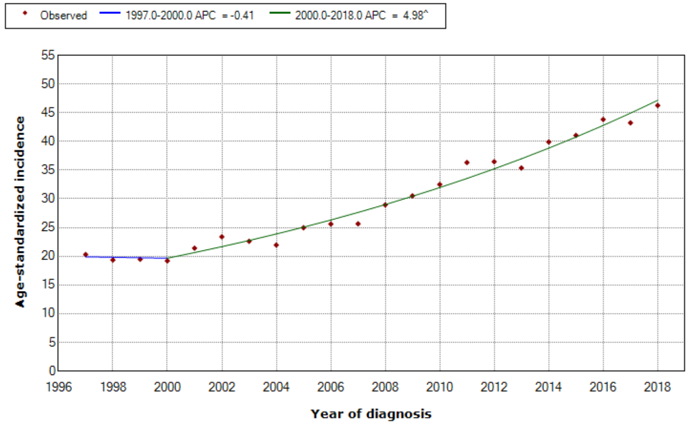

^ Indicates that the Annual Percent Change (APC) is significantly different from zero at the alpha = 0.05 level.
Final Selected Model: 1 Joinpoint.

Missing / Women: 1 Joinpoint

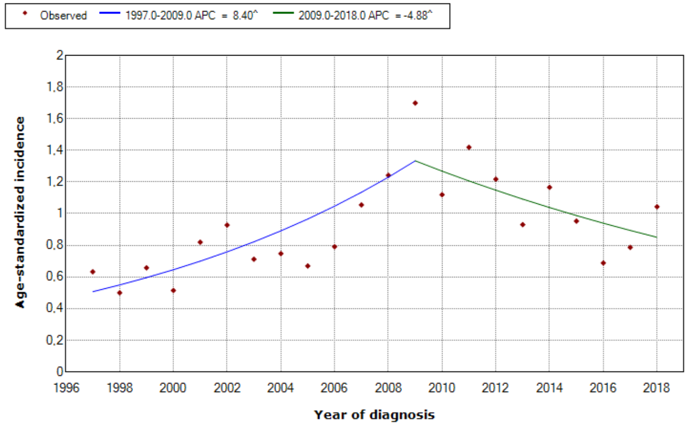

^ Indicates that the Annual Percent Change (APC) is significantly different from zero at the alpha = 0.05 level.
Final Selected Model: 1 Joinpoint.

Missing / Men: 1 Joinpoint

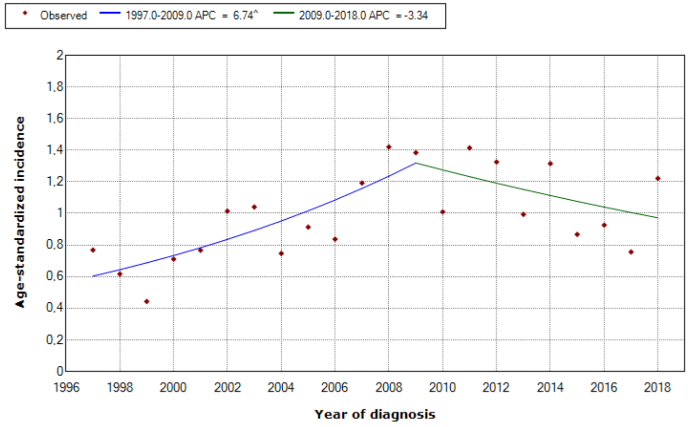

^ Indicates that the Annual Percent Change (APC) is significantly different from zero at the alpha = 0.05 level.
Final Selected Model: 1 Joinpoint.
